# Sex Differences in Gains Among Hispanic Pre-kindergartners’ Mental Rotation Skills

**DOI:** 10.3389/fpsyg.2018.02563

**Published:** 2018-12-17

**Authors:** Carla Abad, Rosalie Odean, Shannon M. Pruden

**Affiliations:** ^1^STEM Transformation Institute, Florida International University, Miami, FL, United States; ^2^School of Education, University of Delaware, Newark, DE, United States; ^3^Department of Psychology, Florida International University, Miami, FL, United States

**Keywords:** spatial thinking, preschool, sex differences, Hispanic children, mental rotation

## Abstract

The current study explores change in mental rotation skills throughout the pre-kindergarten year in a Hispanic population to better understand the development of early sex differences in mental rotation. Ninety-six Hispanic children (*M* = 4 years 8 months) completed a mental rotation task at the beginning and end of pre-kindergarten. Results suggest Hispanic boys and girls differed in gains on mental rotation ability, with boys improving significantly more than girls during pre-kindergarten on a mental rotation task. This study highlights the significance of studying mental rotation abilities in a Hispanic population of pre-kindergarten aged children and suggests the importance of examining sex differences in mental rotation over time, rather than at one time-point, to better understand when sex differences in spatial skills develop. We discuss various factors that potentially affect the growth of spatial skills including the role of early education, spatial experiences, and spatial language input.

## Introduction

Spatial thinking is the ability to think about the spatial world and encompasses a number of skills including mentally rotating and transforming objects and shapes, recreating patterns, and navigating around one’s environment (Sinton et al., 2013). Children and adults depend on spatial thinking for a variety of everyday situations such as remembering the location of a doll in a play room or a car in the parking lot, fitting toys in a box or suitcases in a trunk, and building block towers or Ikea furniture (Abad, 2018). Aside from being necessary for everyday tasks, spatial thinking is linked to early mathematics ability (Cheng and Mix, 2014; Verdine et al., 2017) and predicts future entry in Science, Technology, Engineering, Mathematics (STEM) fields (Humphreys et al., 1993; Shea et al., 2001; Wai et al., 2009).

Several studies have established sex differences in adults’ spatial skills, particularly mental rotation ability, with men consistently outperforming women (Maccoby and Jacklin, 1974; Linn and Petersen, 1985; Voyer et al., 1995). However, when these sex differences develop remains uncertain since sex differences in children’s spatial skills are inconsistent (Frick et al., 2014). Previous studies typically examine sex differences at one timepoint in mostly middle- to upper-income populations of primarily White individuals leaving open the question of whether we see sex differences in spatial thinking over time and in underrepresented populations. The projections that Hispanics will make up 28 percent of the United States population by the year 2050 (Colby and Ortman, 2015) and the lack of minority representation in STEM fields (National Science Foundation and National Center for Science and Engineering Statistics, 2013) reveal the importance of exploring sex differences in a Hispanic population. In a recent publication Levine et al. (2016) attempted to “advance the conversation” on sex differences in spatial cognition by laying out the need for more research examining change in development. The current study seeks to fill these gaps in the literature and “advance the conversation” by examining whether there are sex differences in spatial skills *over time* in a typically understudied population, Hispanic pre-kindergarten (pre-k) children of diverse socioeconomic status. Specifically, the current study aims to explore whether changes in mental rotation ability made by Hispanic boys throughout pre-kindergarten are different from changes made by Hispanic girls.

### Sex Differences in Spatial Thinking

Research over four decades suggests consistent sex differences in spatial thinking, with men reliably outperforming women on some spatial thinking tasks and the largest effects found on tasks requiring mental rotation ability (Maccoby and Jacklin, 1974; Linn and Petersen, 1985; Voyer et al., 1995; Uttal et al., 2013). However, *when* and *how* these sex differences emerge are more contentious subjects (see review by Levine et al., 2016 as well as Frick et al., 2014).

Studies on sex differences in spatial skills across the lifespan have resulted in inconsistent findings. Consistent with the adult literature, some studies have found an early male advantage on spatial skills (e.g., Johnson and Meade, 1987; Levine et al., 1999; Levine et al., 2005; Ehrlich et al., 2006; Casey et al., 2008; Moore and Johnson, 2008; Quinn and Liben, 2008, 2014; Levine et al., 2012; Lauer et al., 2015). For instance, a study by Johnson and Meade (1987) on children between 6 and 18 years showed boys outperform girls on spatial tasks by age 10. Exploring a younger population, Levine et al. (1999) tested children ranging from 4 to almost 7 years of age on a mental rotation task and found sex differences with boys outperforming girls as early as 4.5 years of age. Sex differences on spatial tasks in 3–4.5 year olds have been replicated in other studies (Levine et al., 2012; Joh, 2016; Pruden and Levine, 2017).

However, studies examining children’s spatial thinking have found no consistent sex differences, even on tasks requiring mental rotation skills where the strongest sex differences are found in adults (e.g., Platt and Cohen, 1981; Kaplan and Weisberg, 1987; Caldwell and Hall, 1970; Kaess, 1971; Jahoda, 1979; Kosslyn et al., 1990; Estes, 1998; Lachance and Mazzocco, 2006; Frick et al., 2009; Krüger and Krist, 2009; Jansen and Heil, 2010; Frick et al., 2013; Lehmann et al., 2014; Verdine et al., 2017). For instance, a study by Manger and Eikeland (1998) on sixth graders’ spatial visualization skills found no significant sex differences. Frick et al. (2013) explored the performance of children between the ages of 3 and 5 on a mental rotation task and found no consistent sex differences. More recently, Verdine et al. (2017) assessed the spatial thinking abilities of 3 to 5-year-old children on a variety of spatial tasks and found no significant sex differences. Furthermore, due to the file-drawer problem, it is possible that numerous other studies showing no significant sex differences in children’s spatial thinking remain unpublished (Rosenthal, 1979).

Sex differences in adults’ spatial ability are well established, however, *when* and *how* these sex differences emerge remains uncertain. Biological, hormonal, and evolutionary accounts still permeate the debate, however, environmental factors have been shown to influence and potentially mediate sex differences in spatial skills including: (1) boys are engaged in more activities related to spatial and mathematics achievement than girls (Newcombe et al., 1983; Baenninger and Newcombe, 1995; Nazareth et al., 2013); (2) boys and girls are held to different expectations and standards (i.e., gender stereotypes) by their parents and teachers (Parsons et al., 1982; Eccles and Jacobs, 1986); (3) girls have more anxiety regarding their performance on spatial activities (Lawton, 1994; Baenninger and Newcombe, 1995); and (4) boys hear more spatial language than girls from their parents (Pruden and Levine, 2017).

While no single explanation accounts for the sex differences found in spatial thinking and the timing of the emergence of these sex differences is still debated, it is clear that sex differences exist and are influenced by many environmental factors. The bulk of previous research has addressed sex differences in spatial thinking in middle- to upper-income populations containing primarily non-Hispanic White individuals. However, to better understand sex differences in spatial thinking, it is critical to explore whether these differences exist across populations.

### Generalizability of Sex Differences in Spatial Thinking

Studies investigating whether sex differences in spatial thinking are generalizable across diverse populations show conflicting results. Several studies in African, Asian, and Western cultures suggest the male advantage exists across cultures in both child and adult samples (e.g., Jahoda, 1980; Mann et al., 1990; Lynn, 1992; Silverman et al., 2007; Casey et al., 2008; Lippa et al., 2010; Liu and Lynn, 2011). However, other studies utilizing cross-cultural and diverse populations suggest sex differences in spatial thinking may not be generalizable across all populations. For instance, Feingold (1994) examined sex differences in studies conducted after 1980 with participants from outside of the United States and found no consistent sex differences in verbal, math, or spatial skills across cultures. Berry (1966) examined the spatial ability of Eastern Canadian Eskimos from the Baffin Islands and found no sex differences on a variety of spatial assessments. More recently, Icelandic high school girls were found to outperform their male peers on highly spatial sections of a mathematics test (Lemke et al., 2004). Additionally, socioeconomic status was found to mediate sex differences in mental rotation (Levine et al., 2005). Levine and colleagues found sex differences in the mental rotation skills of boys and girls from middle and high SES but no sex differences in the mental rotation skills of children from low SES.

These studies suggest sex differences in spatial skills are generalizable across some nations, cultures, ethnicities, and socioeconomic statuses but may not be universal. Little research to date (though see Casey et al., 2008; Nazareth et al., 2013) has looked at whether there are similar sex differences in Hispanic individuals across varying socioeconomic groups – the aim of the present study. Importantly, no studies have investigated sex differences in spatial thinking in an exclusively Hispanic population of children within the United States (US). Given the growing Hispanic population in the United States and the current underrepresentation of Hispanic women in STEM fields, it is critical to examine whether sex differences in spatial thinking are generalizable to this particular population.

### Changes in Spatial Skill

Numerous studies have established that spatial thinking is malleable and can be improved through training in both males and females (Ehrlich et al., 2006; Terlecki et al., 2008; Wright et al., 2008). Additionally, studies with multiple timepoints provide a greater understanding of sex differences in spatial thinking by examining whether these differences change over time. However, these studies generally examine change in spatial thinking after a specific intervention, with few studies investigating naturally occurring changes in spatial thinking in males and females throughout development (e.g., Huttenlocher et al., 1998; Levine et al., 2005; Lachance and Mazzocco, 2006; Verdine et al., 2017).

Huttenlocher et al. (1998) conducted a cross-sectional study where kindergartners and first graders were tested during school months and summer break on several cognitive tasks. Emphasizing the impact of early education for the development of spatial skills, children were found to grow significantly more during school months compared to vacation months on cognitive tasks related to language and spatial operations. While this study examines change in spatial thinking throughout early development, its cross sectional design leaves the question of whether boys and girls make similar or different changes throughout the school year unanswered. In a longitudinal study, Levine and colleagues examined the influence of SES and sex on second and third graders’ spatial skills. Children made improvements over time on all spatial tasks measured, however, there were no reported differences in spatial ability by sex or SES over time. A different longitudinal study by Verdine et al. (2017) on the spatial abilities of children between the ages of 3 and 5 found no sex differences in preliminary analysis and therefore did not examine sex differences over time. Another longitudinal study (Lachance and Mazzocco, 2006) where over 100 students were followed from kindergarten to third grade to examine sex differences in math and spatial skills found no persistent sex differences during any year of the study, in any area of math or spatial skills, or in growth rates for math or spatial skills. These studies look at change in spatial skills over time in a naturalistic setting, however, none included an analysis of sex differences in change over time in preschool aged children, a time when many children enter formal schooling and sex differences may be emerging (Levine et al., 1999, 2012; Joh, 2016).

Given the lack of consensus on the age at which sex differences in spatial skills emerge and the fact that spatial skills develop over time and sex differences in spatial skills strengthen over time (Voyer et al., 1995), examining naturally occurring change in boys’ and girls’ spatial skills over time may provide a greater understanding of when these sex differences develop not available through studies with only one timepoint and/or cross-sectional designs. The current study aims to explore sex differences in change on mental rotation throughout the preschool year to better understand the development of early sex differences in spatial skills.

### The Current Study

In sum, prior research finds that sex differences in spatial skills exist (e.g., Maccoby and Jacklin, 1974; Linn and Petersen, 1985; Voyer et al., 1995; Uttal et al., 2013), are generalizable across some populations (e.g., Jahoda, 1980; Mann et al., 1990; Lynn, 1992; Silverman et al., 2007; Casey et al., 2008; Liu and Lynn, 2011), are malleable (e.g., Baenninger and Newcombe, 1989; Uttal et al., 2013), and are influenced by environmental factors (e.g., Newcombe et al., 1983; Baenninger and Newcombe, 1995; Levine et al., 2012; Nazareth et al., 2013). However, little is known regarding change in sex differences in mental rotation ability over time and whether sex differences generalize to an all-Hispanic population. The current study seeks to address this gap by following Hispanic children throughout pre-k to assess early sex differences in mental rotation skills. Specifically, the current study has two aims: (1) to examine whether sex differences in mental rotation skills exist in Fall (time 1) and Spring (time 2) semesters of pre-kindergarten and (2) to explore whether *changes* in mental rotation skills of Hispanic pre-k boys are different from *changes* in mental rotation skills of Hispanic pre-k girls.

## Materials and Methods

### Participants

The sample consisted of 96 children (45 boys; mean age at time 1 = 56 months; *SD* = 3.69 months) from 27 classrooms (20 schools) enrolled in Florida’s state funded pre-k program at private schools. Participants were part of a larger study examining the role of educator language on the development of various spatial skills. One child was excluded due to a diagnosed developmental delay. All children were from Hispanic families in South Florida (16 families or 16.7% refused to report ethnicity) and were Spanish/English bilinguals. An English and Spanish language screener was administered as an additional check that they were being raised in bilingual homes (see section “Materials’ and Methods” for a description of the language screener). Socioeconomic status (SES), which has been shown to mediate sex differences in spatial skills (Levine et al., 2005) was diverse, with families reporting variability in two indicators of SES, income and education levels. Given the correlation between family gross income and highest degree of education, *r*(94) = 0.630, *p* < 0.001, gross income was used as a proxy for SES. Eight participants reported earning $100,000 or more a year (8.3%), 5 earning between $75,000 to $99,999 (5.2%), 19 earning between $50,000 and $74,999 (19.8%), 13 earning between $35,000 and $49,999 (13.5%), 18 earning between $15,000 and $34,999 (18.8%), and 13 earning less than $15,000 a year (13.5%). Twenty families did not report gross income (20.8%).

### Materials and Procedures

#### Consent and Demographics

Participants were recruited via letters sent from schools to parents. Interested families returned a signed consent form and received a demographics questionnaire regarding the child’s race and ethnicity, primary caregiver’s highest level of education, and family gross income.

#### Language Comprehension Screener

Children with parental consent were administered a brief language comprehension task in both English and Spanish during the first school visit. The screener included five questions in English (i.e., *what is your name, point to the cat, how old are you, show me your nose, what is your favorite color*) and Spanish (i.e., *cómo te llamas, señala el gato, cuántos años tienes, enseñame tu nariz, cuál es tu color favorito*) intended to assess basic language comprehension in both languages. Children were tested in a random order and the first two boys and two girls from each classroom to answer a minimum of four out of five items correctly on both language screeners were included in the study. Only four children from each classroom were selected to ensure a balanced number of children from each classroom and to limit classroom disruption. Forty-four children were excluded from the study for failing the screener in either English or Spanish. This confirmed children were able to understand English for our English-administered mental rotation assessment and were bilingual.

#### Assessments

Participants completed the *Peabody Picture Vocabulary Test, Test de Vocabulario en Imágenes Peabody*, and *Children’s Mental Transformation Task* at both Fall and Spring semesters. The average time lag between time 1 and time 2 assessments was approximately four and a half months. Children were tested individually at their preschool and were given a sticker at the end of each testing session as a reward.

##### Receptive vocabulary

Children completed a measure of receptive vocabulary in English (*Peabody Picture Vocabulary Test, 4th ed* [PPVT]; Dunn and Dunn, 1997) and Spanish (*Test de Vocabulario en Imagenes Peabody* [TVIP]; Dunn et al., 1986) twice during the school year. These measures served as a proxy of children’s verbal intelligence; standardized scores for both the PPVT and TVIP were not significantly correlated [*r*(94) = -0.195, *p* = 0.07] and were both included as control variables in analyses. Since bilingual children vary in their relative strength in each language spoken, we found it to be important to include both measures to accurately represent children’s vocabulary skills. For each test item, the experimenter asked the child to point to a picture from a set of four pictures (e.g., “point to feather”). Each assessment took approximately 10–15 min to administer. Scores on both the PPVT and TVIP were age-based standardized scores with a mean score of 100 and a standard deviation of 15.

##### Children’s mental transformation task

**FIGURE 1:**
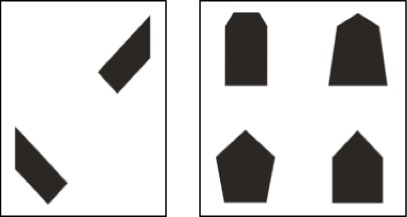
Example from the Children’s Mental Transformation Task (CMTT). Children are shown two pieces of shapes and four shapes and are asked to “*Point to the shape the pieces make*.”

An abbreviated version of the *Children’s Mental Transformation Task* (CMTT; Levine et al., 1999) used by Pruden et al. (2011) was administered at each timepoint. This task evaluates children’s ability to mentally rotate and translate two shapes to make a whole object. The CMTT is different from classic embedded figures task in that it requires both rotation and transformation of object parts to form a whole rather than simply identifying parts within a whole. On each of 10 items, children were shown two pieces of shapes and four target shapes, and were asked to point to the shape that the two pieces would make if they were put together (Figure 1). Every correct response received 1 point with a possible score range of 0 to 10 points. On average, the CMTT took 5 min to complete and children were administered all 10 items.

## Results

### Normality, Outliers, and Missing Data

Prior to analysis, SES, child’s age at the time of each assessment, and scores on the PPVT, TVIP, and CMTT at each timepoint were examined for normality as well as univariate and multivariate outliers. Histograms were examined for univariate outliers and violations of normality (Tabachnick and Fidell, 2013). No univariate outliers were found in any variables tested. However, several variables were found to be skewed; this was addressed by using bootstrapping in subsequent analyses. No multivariate outliers were identified by using Mahalanobis distance with *p* < 0.001 (Yuan and Hayashi, 2010). Little’s Missing Completely At Random (MCAR) test was not significant (*X*^2^= 767.92, *df* = 816, *p* = 0.884) suggesting data were missing at random (Tabachnick and Fidell, 2013). Less than 13% of data were missing, missing data were addressed by conducting multiple imputations using five imputations (Schafer and Graham, 2002). Reported results are from analyses conducted utilizing pooled data from the five imputations.

### Descriptive Statistics

Descriptive statistics for children’s performance on the CMTT and the receptive vocabulary measures (PPVT; TVIP) show considerable variability at each timepoint and in both sexes (Table 1). Average assessment scores suggest no floor or ceiling effects for any of the assessments, and ranges suggest variability in children’s performance.

**Table 1:** Descriptive statistics for scores at time 1 and time 2.

	Time 1						Time 2					
					Males	Females					Males	Females
	***M***	***SD***	**Min**	**Max**	***M* (SD)**	***M* (SD)**	***M***	***SD***	**Min**	**Max**	***M* (SD)**	***M* (SD)**
CMTT	4.04	2.54	0.00	9.00	4.45 (2.43)	3.68 (2.61)	5.69	2.07	0.00	10.00	5.58 (2.43)	5.78 (1.72)
PPVT	89.60	14.74	59.00	117.00	87.92 (15.14)	91.23 (14.34)	93.79	15.06	59.00	120.00	94.31 (14.58)	93.36 (15.60)
TVIP	90.23	17.58	55.00	135.00	90.21 (16.41)	90.25 (18.73)	91.13	19.66	55.00	139.00	90.71 (18.79)	91.47 (20.56)

*Scores in this table were not imputed.*

### Main Analyses

Two multiple linear regressions and an ANCOVA style linear regression were conducted with Mplus version 7.31 to assess whether child sex was predictive of performance on the CMTT at time 1 and time 2 (aim 1) and to examine whether child sex was predictive of *changes* in CMTT scores throughout pre-kindergarten (aim 2).

Aim 1. A multiple linear regression was performed at each of the two timepoints to examine whether there were sex differences in mental rotation scores at time 1 (see Table 2) and time 2 (see Table 2); each regression controlled for SES, age at the time of assessment, and receptive vocabulary (PPVT and TVIP) scores. Results suggest there were no significant sex differences in Hispanic pre-kindergartners’ CMTT scores at the time 1 (CMTT: *b* = -0.65, β = -0.13, *p* = 0.18, *R*^2^ = 0.201) or time 2 (CMTT: *b* = 0.28, β = 0.07, *p* = 0.51, *R*^2^ = 0.134). Given the bilingual population of this study, English and Spanish receptive vocabulary scores were both included as controls; however, the results held when controlling for only English and only Spanish scores.

**Table 2:** Regression analyses predicting CMTT at time 1 for imputed pooled scores.

	Time 1				Time 2			
Variable	*B*	SE (B)	β	*p*	*B*	SE (B)	β	*p*
Constant	-17.89	3.49	-7.07	0.00	-8.51	4.29	-4.06	0.05
Child’s sex	-0.65	0.49	-0.13	0.18	0.28	0.43	0.07	0.51
Family income	-0.07	0.17	-0.05	0.66	0.23	0.17	0.18	0.18
PPVT	0.04	0.02	0.24	0.05^∗^	0.03	0.02	0.20	0.13
TVIP	0.02	0.01	0.14	0.16	0.02	0.01	0.15	0.12
Age	0.23	0.06	0.32	0.00^∗∗^	0.07	0.06	0.11	0.26

*Time 1 R^2^ = 0.201; Time 2 R^2^ = 0.134; ^∗^p < 0.05 and ^∗∗^p < 0.01.*

Aim 2. An ANCOVA style linear regression was performed on CMTT scores (see Table 3); ANCOVA style linear regressions can be used to determine change with two timepoints by using statistical controls to investigate change over time (Newsom, 2012). An ANCOVA style regression allows for similar conclusions as an ANOVA while permitting the inclusion of additional continuous variables to be controlled (Cohen et al., 2003).

**Table 3:** ANCOVA analyses predicting CMTT at time 2 for imputed pooled scores.

Variable	*B*	SE (B)	β	*p*
Constant	-0.488	4.326	-0.237	0.910
Time 1 score	0.410	0.162	0.496	0.011^∗^
Child’s sex	0.431	0.433	0.102	0.320
Child’s sex ^∗^ time 1	-0.408	0.177	-0.367	0.021^∗^
Family income	0.191	0.150	0.149	0.203
PPVT	0.023	0.017	0.178	0.179
TVIP	0.010	0.010	0.089	0.348
Age	0.040	0.060	0.068	0.502

*R^2^ = 0.234; ^∗^p < 0.05. CI, confidence interval; LL, lower limit; UL, upper limit.*

**FIGURE 2:**
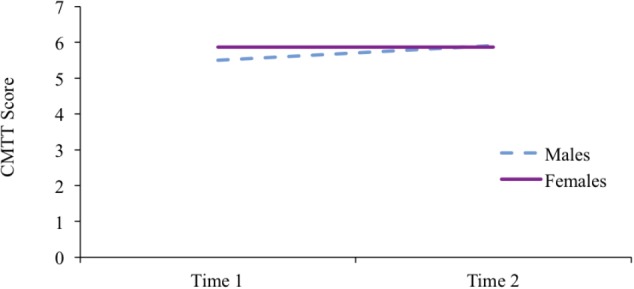
Gains in CMTT scores throughout pre-kindergarten by child’s sex based on regressions including age at the time of assessment, family gross income, and English and Spanish receptive vocabulary scores as covariates with mean covariates values.

The second aim of the study was to explore whether there are sex differences in the *changes* Hispanic children make in mental rotation skills during prekindergarten. Results show that the interaction between child’s sex and CMTT scores at time 1 significantly predicted CMTT scores at time 2 (*b* = -0.41, β = -0.37, *p* = 0.02, *R*^2^ = 0.234), suggesting there were significant sex differences in *gains* made throughout pre-k on this task. Specifically, boys improved 0.41 points more than girls on the CMTT throughout the school year. English and Spanish receptive vocabulary scores were both included as controls; however, results held when controlling for only English or only Spanish scores. These results suggest boys are experiencing significantly greater gains than girls on the CMTT (see Figure 2). Furthermore, CMTT scores at time 1 were found to be a significant positive predictor of CMTT scores at time 2 (*b* = -0.41, β = 0.50, *p* = 0.011, *R*^2^ = 0.234), suggesting improvement in CMTT scores throughout pre-k when boys and girls scores are combined.

It is important to note that results from the ANCOVA style linear regressions seem to contradict descriptive statistics previously reported, where the mean score on the CMTT for girls increased more than the mean score on the CMTT for boys across timepoints (Table 1); however, simply looking at mean scores does not take into account individual differences in performance. In fact, examining standard deviations for mean scores shows that while there is a similar amount of variability in boys’ and girls’ scores at time 1, there is much greater variability in boys’ scores compared to girls’ scores at time 2. Furthermore, simply comparing mean scores does not control for any potential confounds. The literature points to SES, age, and verbal IQ as strong predictors of CMTT scores (e.g., Levine et al., 2005), therefore, in order to accurately assess growth in CMTT it is necessary to control for these variables. Since our control variables are continuous variables, but we are interested in change over time and sex differences, which are categorical variables, ANCOVA, which within the general linear model combines ANOVA and regression, is the most appropriate and least biased approach (see Rutherford, 2011 for a detailed explanation of the uses of ANCOVA). For these reasons, an ANCOVA style regression was run in order to gain a more complete understanding of the development of mental rotation skills throughout pre-k.

## Discussion

The current study aimed to examine the development of sex differences in the mental rotation skills of Hispanic children throughout pre-k by exploring (1) whether sex differences exist in Hispanic pre-kindergartners’ mental rotation skills at time 1 and time 2; and (2) whether there are sex differences in the *changes* (i.e., *gains*) Hispanic children make on mental rotation skills throughout pre-k. In short, our results suggest that simply looking at sex differences at only one time would suggest there are no sex differences in mental rotation skills at this age; however, by examining sex differences over time with robust analysis utilizing potential covariates, boys were found to make greater gains in mental rotation than girls. The importance of these findings is threefold as they suggest (1) pre-kindergarten is a time of significant change and emergence of sex differences in mental rotation skills; (2) different methodologies such as including multiple timepoints and examining change is critical for understanding when sex differences in mental rotation skills develop; and (3) early sex differences in mental rotation are generalizable to a SES-diverse population of Hispanic children living in the United States.

### Sex Differences in Mental Rotation Skills

Our findings that boys make greater gains in mental rotation than girls throughout prekindergarten provide interesting insight into the study of sex differences in childhood. While sex differences in the mental rotation skills of adults are well established, studies examining sex differences in children’s spatial skills show inconsistent results (Frick et al., 2014). Our results help explain these discrepancies in the literature, as they show pre-k boys make greater improvements than girls on a task requiring mental rotation skills, but boys and girls do not significantly differ in performance when examined at one single timepoint. Furthermore, while there is a similar amount of variability in boys’ and girls’ scores at time 1, there is greater variability in boys’ scores compared to girls’ scores at time 2. These findings suggest that pre-kindergarten is a time when sex differences in mental rotation skills are emerging, though more studies will be needed to determine when exactly these differences emerge.

One possible explanation for boys improving significantly more on the CMTT than girls is that early education may be providing more opportunities for boys, compared to girls, to advance their mental rotation skills. Previous research shows boys are exposed to more activities (e.g., Legos, blocks, construction toys) that promote spatial learning than girls (Newcombe et al., 1983; Baenninger and Newcombe, 1995; Nazareth et al., 2013). While speculative, since we did not gather data on children’s exposure to spatial activities in the classroom, it is possible boys may have been exposed to new spatial experiences that improved their mental rotation skills in pre-k, allowing them to make larger gains on the CMTT. Another possible explanation for boys improving more than girls on the CMTT is that boys hear more spatial language than girls from their parents in the home setting (Pruden and Levine, 2017). Similarly, it could be that educators, like parents, use new or a greater quantity and quality of spatial language with boys in the pre-k classroom, contributing to the development of boys’ spatial skills. While speculative, these various factors highlight the need for more research on spatial development in the early education setting, the impact of early education on spatial development, and whether boys and girls are exposed to the same kind of spatial experiences (e.g., activities and language) in the early education classroom (Costales et al., 2015). These mechanisms alone, or more likely via complex interactions, may provide a powerful means to promote spatial thinking in both sexes.

### Examining Sex Differences at Multiple Timepoints

The current findings suggest the importance of examining change in sex differences in mental rotation through time to better understand these differences and when and how they develop. Had we explored sex differences at only one of the two timepoints, we would have concluded that there were no sex differences in Hispanic pre-kindergartners’ mental rotation skills. However, by examining change in boys’ and girls’ mental rotation skills throughout the school year, we were able to observe that mental rotation ability in pre-k is actually different for boys and girls, with boys making greater improvement on a mental rotation task than girls.

Given our finding that sex differences in mental rotation are growing stronger or emerging at this age, it is possible that studies looking for sex differences at only one timepoint may not always find significant results, even when differences are present. Our results highlight the importance of utilizing different methodologies examining change over time to uncover the earliest indicators of sex differences in spatial thinking and help explain the inconsistencies in the current research on early sex differences in spatial thinking. Given that spatial skills are malleable and can be improved (Uttal et al., 2013), early detection of sex differences in spatial thinking by exploring change is critical and may help us identify when in development spatial experiences and training for both girls and boys should occur.

### Generalizing Sex Differences in Mental Rotation to a Hispanic Population

Studies exploring sex differences in diverse populations show inconsistent results (Berry, 1966; Jahoda, 1980; Mann et al., 1990; Lynn, 1992; Feingold, 1994; Lemke et al., 2004; Levine et al., 2005; Silverman et al., 2007; Casey et al., 2008; Liu and Lynn, 2011). To date, few studies have examined sex differences with diverse populations including Hispanic participants (though see Casey et al., 2008; Nazareth et al., 2013). Our findings suggest sex differences in mental rotation skills are generalizable to a Hispanic population of children of varying SES living in the United States. Given the link between spatial thinking and future entry into STEM fields (Humphreys et al., 1993; Shea et al., 2001; Wai et al., 2009) and the exponentially increasing Hispanic population in the United States, our findings may have important implications for future work aimed at understanding the underrepresentation of minorities and women in STEM fields.

### Limitations

It is important to note some limitations to this study. First, the current study only measured mental rotation ability at two timepoints, limiting our ability to test non-linear relations and utilize more powerful statistical tools like growth curve modeling. Second, given the small number of children assessed at some of the classrooms and schools, it was not possible to determine whether there were any classroom or school clustering effects. Third, given the observational nature of this study, we are unable to make causal inferences regarding the mechanisms that lead to changes in mental rotation performance and the sex differences seen in changes on the mental rotation task. Fourth, given the nature of this study it was not possible to control for the influence of many factors which have been shown to influence sex differences in mental rotation skills (e.g., engagement with spatial toys and activities, gender stereotypes, spatial anxiety, and spatial language. Finally, our study is the first to examine spatial thinking skills in a bilingual population. While we believe this is an important and understudied area of research, given that our entire sample was English-Spanish bilinguals, it was not possible for us to examine the specific role of bilingualism versus monolingualism on the development of spatial thinking.

## Conclusion

The current study suggests there are sex differences in the gains made throughout pre-k on mental rotation, with boys making significantly more gains than girls. The current findings point to the need to explore change over time to attain a greater understanding of sex differences in mental rotation ability. Future research should continue to explore the influence of early schooling on the development of spatial skills in diverse populations, with a particular focus on the mechanisms resulting in changes in spatial thinking and the factors leading to sex differences in these changes (e.g., spatial activities and spatial language). Given the link between spatial thinking and future entry into STEM fields (Humphreys et al., 1993; Shea et al., 2001; Wai et al., 2009), a better understanding of the influence early education, among other potential factors, on spatial development in boys and girls from diverse backgrounds is needed. Identifying mechanisms that promote growth in spatial thinking is critical to increasing the number of minorities and women entering STEM fields.

## Ethics Statement

This study was carried out in accordance with the recommendations of Florida International University’s Institutional Review Board. Participants’ primary caregivers gave written informed consent in accordance with the Declaration of Helsinki. The protocol was approved by the Institutional Review Board at Florida International University.

## Author Contributions

CA, RO, and SP contributed to conception and design of the study. CA and RO collected the data and performed the statistical analysis. CA wrote the first draft of the manuscript. All authors contributed to manuscript revision, read and approved the submitted version.

## Conflict of Interest Statement

The authors certify that they have NO affiliations with or involvement in any organization or entity with any financial interest (such as honoraria; educational grants; participation in speakers’ bureaus; membership, employment, consultancies, stock ownership, or other equity interest; and expert testimony or patent-licensing arrangements), or non-financial interest (such as personal or professional relationships, affiliations, knowledge, or beliefs) in the subject matter or materials discussed in this manuscript.
